# Commonalities and Asymmetries in the Neurobiological Infrastructure for Language Production and Comprehension

**DOI:** 10.1093/cercor/bhab287

**Published:** 2021-09-07

**Authors:** Laura Giglio, Markus Ostarek, Kirsten Weber, Peter Hagoort

**Affiliations:** Max Planck Institute for Psycholinguistics, 6525 XD Nijmegen, The Netherlands; Donders Institute for Cognition, Brain and Behaviour, Radboud University, 6525 AJ Nijmegen, The Netherlands; Max Planck Institute for Psycholinguistics, 6525 XD Nijmegen, The Netherlands; Donders Institute for Cognition, Brain and Behaviour, Radboud University, 6525 AJ Nijmegen, The Netherlands; Max Planck Institute for Psycholinguistics, 6525 XD Nijmegen, The Netherlands; Donders Institute for Cognition, Brain and Behaviour, Radboud University, 6525 AJ Nijmegen, The Netherlands; Max Planck Institute for Psycholinguistics, 6525 XD Nijmegen, The Netherlands; Donders Institute for Cognition, Brain and Behaviour, Radboud University, 6525 AJ Nijmegen, The Netherlands

**Keywords:** syntax, speaking, sentence, fMRI, constituent structure

## Abstract

The neurobiology of sentence production has been largely understudied compared to the neurobiology of sentence comprehension, due to difficulties with experimental control and motion-related artifacts in neuroimaging. We studied the neural response to constituents of increasing size and specifically focused on the similarities and differences in the production and comprehension of the same stimuli. Participants had to either produce or listen to stimuli in a gradient of constituent size based on a visual prompt. Larger constituent sizes engaged the left inferior frontal gyrus (LIFG) and middle temporal gyrus (LMTG) extending to inferior parietal areas in both production and comprehension, confirming that the neural resources for syntactic encoding and decoding are largely overlapping. An ROI analysis in LIFG and LMTG also showed that production elicited larger responses to constituent size than comprehension and that the LMTG was more engaged in comprehension than production, while the LIFG was more engaged in production than comprehension. Finally, increasing constituent size was characterized by later BOLD peaks in comprehension but earlier peaks in production. These results show that syntactic encoding and parsing engage overlapping areas, but there are asymmetries in the engagement of the language network due to the specific requirements of production and comprehension.

Since the association of lesions in the left inferior frontal gyrus (LIFG) and aphasia in the nineteenth century, scientists have tried to understand the relationship between the language faculty and the brain. Early reports called the LIFG or Broca’s area a “speech movement centre” and the left superior temporal gyrus (LSTG) or Wernicke’s area a “sensory speech centre” (from Wernicke 1892, as described in Levelt 2013). Since then, the field moved forward from a production-comprehension dissociation to the understanding that both areas are critical for language more generally, and that they do not subserve strictly segregated receptive or productive linguistic functions (Tremblay and Dick 2016). A wealth of neuroimaging studies and lesion-symptom mapping studies advanced the characterization of brain function greatly, which resulted in a general understanding of the contributions of core regions in the language network (e.g., Friederici and Gierhan 2013; Hagoort and Indefrey 2014; Price 2012; Wilson 2017).

The LIFG (i.e., Broca’s area and adjacent cortex) has been implicated in sentence-level processes in several neuroimaging studies. These included sentence vs. word list comprehension (Snijders et al. 2009; Fedorenko et al. 2012; Matchin et al. 2017; Zaccarella, et al. 2017b), phrase structure building (Schell et al. 2017; Zaccarella, et al. 2017a; Pallier et al. 2011; Chang et al. 2020), compositional processes in naturalistic language comprehension (Henderson et al. 2016; Bhattasali et al. 2019), and processing of noncanonical sentence structure (Bornkessel-Schlesewsky et al. 2009; Santi and Grodzinsky 2010; Hirotani et al. 2011; Mack et al. 2013; Europa et al. 2019). In different neurobiological models of language processing the LIFG was thus proposed to have a role in combinatorial (Unification) processes in multiple domains of language and cognition (Hagoort 2005; Hagoort 2013; Hagoort 2019; Jackendoff and Audring 2020); in processing complex syntax (Friederici 2012); or in sentence processing due to its role in working memory (Matchin, 2018; Rogalsky et al. 2008).

Within the temporal lobe, posterior regions have been implicated in several aspects of comprehension, from auditory to phonological and morphological processing along the superior temporal gyrus and sulcus (Hickok and Poeppel 2007; Friederici 2012; Lee et al. 2018). In addition, the posterior middle temporal gyrus (LpMTG) has been associated with the retrieval of lexical-syntactic frames (“Memory” processes, Hagoort, 2005, 2013) and syntactic processes (Flick and Pylkkänen 2020; Matchin and Hickok 2020). The anterior temporal lobe (ATL) has been associated with conceptual operations (e.g., Bemis and Pylkkänen 2013; Boylan et al. 2017), also based on findings of ATL atrophy leading to semantic dementia (Wilson et al. 2013; Mesulam et al. 2014; Lambon Ralph et al. 2017).

All studies mentioned above, however, are based on linguistic processes in *comprehension*. The involvement of the main nodes of the language network in sentence *production* is less clear. This is mainly for two reasons: (i) the challenge of achieving good experimental control in sentence production studies, which also limited psycholinguistic studies of production processes (Bock 1996), and (ii) the obstacle of motion artifacts in neuroimaging as a consequence of movement during speech, which is however not impossible to overcome with state-of-the-art neuroimaging techniques (Willems and van Gerven 2018). These methodological difficulties have led to far fewer studies on the characterization of brain involvement in production than comprehension. As a consequence, the few meta-analyses that attempted to characterize the language network in the two modalities were severely underpowered in production (Walenski et al. 2019; Indefrey 2018).

The network obtained by meta-analyses of sentence production studies does not fully or consistently overlap with the sentence comprehension network discussed above. Sentence production studies found activity within a left-lateralised fronto-temporal network but not consistently across studies (Indefrey et al. 2001; Indefrey et al. 2004; Haller et al. 2005; Kircher et al. 2005; Golestani et al. 2006; den Ouden et al. 2008; Grande et al. 2012; Collina et al. 2014; Humphreys and Gennari 2014; Pylkkänen et al. 2014; Thothathiri and Rattinger 2015; Matchin and Hickok 2016; Thothathiri 2018; Takashima et al. 2020). A recent meta-analysis on some of these studies found left middle frontal gyrus, LpMTG and lateral occipital cortex to be reliably involved in sentence production, but did not find evidence for LIFG involvement (Walenski et al. 2019). Another meta-analysis, instead, found the LIFG to be the only area reliably active in sentence production and for syntactic contrasts across some of those studies, thus lacking temporal lobe involvement (Indefrey 2018). There is thus disagreement on whether inferior frontal areas or temporal areas are reliably engaged during sentence production, while they are both reliably found in sentence comprehension. The contradictory results of these meta-analyses show that more work is needed to robustly determine the neural correlates of sentence production. Interestingly, these results suggest there are some discrepancies in the networks engaged by linguistic processes in production and comprehension that raise the question whether the same neural resources are used in production and comprehension.

An important line of work addressed the question of a shared or distinct neural infrastructure between sentence production and comprehension. This question is relevant in the context of a long-standing debate in psycholinguistics. There are different views on if, and to what degree, production and comprehension share phonological, lexical, syntactic and semantic representations (Meyer et al. 2016; Momma and Phillips 2018; Phillips 2013; Gambi and Pickering 2017). Support for distinct representations comes from the production/comprehension asymmetries in language acquisition. Comprehension is seen to precede production in many linguistic domains, with some exceptions (Clark and Hecht 1983; Hendriks and Koster 2010). This dissociation in acquisition is more easily accounted for by models that keep production and comprehension representations separate. Accounts of syntactic deficits in agrammatic patients also suggest that different processes are compromised in comprehension and production (i.e., tree pruning in production vs. trace deletion in comprehension) (Grodzinsky 2000). However, there are also views supporting a single processing mechanism that argue that the differences between modalities may be superficial and may instead reflect input differences (Momma and Phillips 2018). Behavioral evidence has shown that syntactic representations are shared between production and comprehension (Kempen et al. 2012). Also, repetition suppression (Grill-Spector et al. 2006) was used in fMRI to understand which areas adapt to the repetition of linguistic material and whether the adaptation occurs only within one language modality or also across modalities (i.e., from comprehension to production and vice versa). The LIFG, precentral gyrus, LMTG and inferior parietal lobule were found to adapt to syntactic and lexical repetition across sentence production and comprehension, suggesting that production and comprehension share neural resources (Menenti et al. 2011; Menenti et al. 2012; Segaert et al. 2012; Segaert et al. 2013). This evidence for shared resources in production and comprehension is, however, challenged by the inconsistent and partly contradictory neuroimaging results in production.

In the current study, therefore, we examined the sentence production network in a high-powered study with the aim to further clarify the brain organization of sentence production. To address this issue, our study investigated language production in analogy to a seminal study on constituent structure building in comprehension (Pallier et al. 2011). Constituents are the syntactic building blocks of sentences. By using a constituent size manipulation, we could focus on the processes that allow for encoding of increasingly larger structures, while keeping lexico-semantic, phonological and articulatory processes constant between conditions. Following Pallier et al. (2011), we expected neural activity to gradually increase with the addition of each new node to the constituent structure of the stimuli. We used visual prompts to elicit the production of utterances that had three levels of constituent structure which differed in complexity. The simplest one consisted of one- and two-word sequences; the intermediate condition consisted of intransitive sentences; the version with the most complex structure had participants produce a sentence with a complementizer phrase embedded in the main clause. In their comprehension study, Pallier et al. (2011) showed that LIFG and the left anterior and posterior temporal lobe were responsive to constituent size. Based on previous comprehension evidence we therefore expected to find a gradual involvement of at least LIFG and LMTG with increasing constituent size.

Additionally, we directly compared the sentence production and comprehension networks with the aim to further clarify to what extent they overlap. Few studies so far used both production and comprehension in the same experiment, including a direct comparison between modalities. In particular, it is still unclear whether sentence production and comprehension rely on core regions of the language network to the same or to a different extent. Humphreys and Gennari (2014) found that frontal and subcortical regions were more engaged in production, while the LpMTG was more engaged in comprehension. Indefrey et al. (2004) found the LIFG to be responsive to syntactic processing in production but not in comprehension. Matchin and Wood (2020) instead found similar activity in LIFG for syntactic production and comprehension, and larger activity in LMTG for syntactic comprehension than production. We therefore selected LIFG and LMTG as regions of interest to better characterize their involvement in sentence processing across modalities. In short, there is no clear answer to the question whether production and comprehension recruit frontal and temporal regions similarly or differently. In this study we attempted to answer this question.

**Table 1:** Example sentences used for each condition

Condition	Stimuli (in Dutch)	English translation
C1	klappen, slapen, de jongen, het meisje	clap, sleep, the boy, the girl
C2	de jongen slaapt, het meisje praat	the boy sleeps, the girl talks
C4	de jongen hoort dat het meisje klapt	the boy hears that the girl claps
Filler	de man helpt de vrouw	the man helps the woman

## Materials and Methods

### Participants

Forty-six right-handed native Dutch participants (28 females, mean = 23.8 years, range 19–35 years) participated in the experiment in return for monetary compensation after giving written informed consent. The study was approved by the ethical committee for Region Arnhem-Nijmegen. Participants had no history of neurological or language-related disorders, and reported having normal or corrected-to-normal vision and hearing. Six participants were excluded for the following reasons: technical problems during preprocessing of the MRI data (n = 1); failing to complete the experiment (n = 2); too many motion artifacts (n = 3). Forty participants were included in the analyses. This number was based on an *a priori* power calculation for the detection of an effect for the production of passive vs. active sentences in the LIFG and LMTG in a previous study (Segaert et al. 2012), using fMRIpower (Mumford and Nichols 2008). Even though the specific manipulation was different, it allowed us to estimate the number of participants needed for the detection of a syntactic effect in production in the two regions of interest.

### Materials

In our study, we had three levels of constituent structure (see Table 1). The condition with the smallest constituent size (C1) consisted of two verbs and two noun phrases leading to four constituents with one (content) word (C1: “klappen, slapen, de jongen, het meisje”, “to clap, to sleep, the boy, the girl”). The intermediate condition (C2) involved the combination of a verb and a noun phrase leading to two constituents with two content words forming intransitive sentences (C2: “de jongen slaapt, het meisje praat”, “the boy sleeps, the girl talks).^1^ The most complex sentence condition (C4) consisted of the combination of the four content words into a complementizer phrase embedded in the main clause (C4: “de jongen hoort dat het meisje klapt”, “the boy hears that the girl claps”). Critically, the conditions were almost identical in the total number of words to be produced, but they differed in constituent structure. The embedded sentence condition included the additional word “dat” (*that*), which in Dutch is obligatory in complementizer sentences.^2^ We did not expect function words to affect sentence planning but they might involve articulation-related processes (Ferreira 1991). An additional filler condition was added to avoid too many verb repetitions. Filler sentences consisted of a sentence with one transitive verb (“de man helpt de vrouw”, “the man helps the woman”).

**Figure 1:**
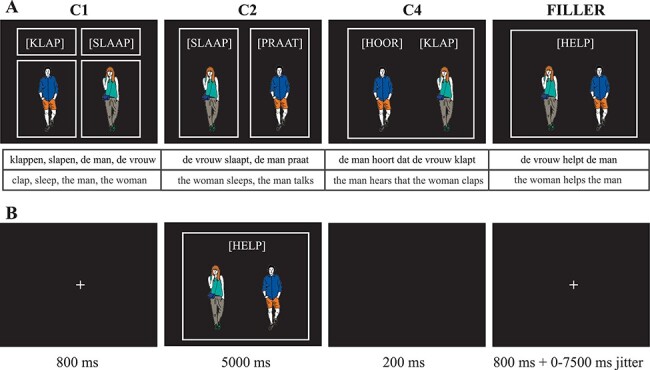
Stimulus presentation. **A**: Example of the screen that participants would see for each condition (identical in production and comprehension) with the corresponding expected output. The boxes clarified the type of output that was required. **B**: Screen sequence for each trial. The length of the fixation cross presentation was based on jittering optimized for contrast detection. In comprehension, during picture and verb presentation, a sound recording of the sentence started after 1000 ms.

To induce the production of the sentences in the different conditions, participants were shown pictures with written verbs (see Fig. 1). Crucially, the conditions differed in the configuration of boxes around the verbs and the pictures of human figures. The boxes instructed the participants about the production output that was expected. In condition C1, there were four boxes, one around each item, signaling that the production of four separate items was expected. In this condition the actors and verbs should not be combined to form a sentence. In condition C2, there were two boxes, each around a verb and an actor, indicating that two separate sentences had to be produced. In condition C4, there was one box around all items on the screen, indicating that one single sentence was expected with the first verb heading an embedded clause formed by the second noun and verb. For filler sentences, there was only one box around all items on the screen. In this case there was only one verb, indicating that a transitive sentence was expected. Participants had no problems understanding the task and producing the correct output. By eliciting sentence production in this way, we could minimize the visual differences between conditions: pictures or videos would lead to very large differences in the visualization of word sequences vs. complementizer phrases. This type of speech elicitation paradigm is not unusual in the neuroimaging sentence production literature (cf. Matchin and Hickok 2016; Takashima et al. 2020).

The verbs were always presented in their root form, so that the production of the syntactically correct inflections was required in all conditions. In the C1 condition, the verb had to be produced in its infinitival form (generally, by addition of “en”: *help* to *helpen*); in the other conditions, the verb had to be inflected in the third person singular of the present tense (generally, by addition of “t”: *help* to *helpt*).

Since verbs allowing for a complementizer phrase (CP-verbs) and intransitive verbs (INT-verbs) are inherently different in their use and meaning, we selected a few verbs of each type that were repeated 8 times across the experiment. The verbs were matched in frequency (mean ± std: INT-verbs = 1.38 ± 0.88, CP-verbs = 1.46 ± 0.77, *t* = 0.59, *p* = 0.56) based on SUBTLEX-NL values (Keuleers et al. 2010), and concreteness (mean ± std: INT-verbs = 3.26 ± 0.67, CP-verbs = 3.21 ± 0.47, *t* = 0.27, *p* = 0.79) (Brysbaert et al. 2014). Each condition consisted of 80 trials. In C4, we used 20 CP-verbs, repeated 4 times. The CP-verbs were always in first position to allow for the embedded sentence production; each of the 40 INT-verbs in C4 was repeated twice. In C2, we used the same 40 INT-verbs, each presented twice in first position and twice in second position. In C1, there was always one CP-verb and one INT-verb, with alternating first and second positions. Each CP-verb was repeated 4 times in this condition, and each INT-verb was repeated twice. The filler verbs consisted of 80 transitive verbs, each shown only once. We created 4 lists of stimuli that consisted of the same verb combinations for each condition, but for each list the verb was paired with a different picture. Across lists each verb combination was paired with each actor. The actors could be “the boy”, “the girl”, “the man”, “the woman”, with each presented 160 times in total.

In addition to the production condition, we included a comprehension condition that included half of the materials used for production but from a different list (hence with different actor-verb pairings). In the comprehension condition, each verb was repeated only 4 times in total, with 40 trials per condition. Instead of producing the sentences, participants had to listen to recorded stimuli, which started 1 sec after picture onset and lasted a maximum of 4 seconds (mean duration (in seconds): C1 = 3.14, C2 = 2.46, C4 = 2.46, Fillers = 1.79). The absence of an explicit task during the comprehension runs kept the production and comprehension runs as similar as possible without the introduction of effects unrelated to constituent size.

### Experimental Procedure

The experiment started with a behavioral practice session to familiarize participants with the task. They read instructions for each condition and had to practise producing the sentences. The experimenter gave feedback to make sure that the participant understood the task correctly. After the practice session was concluded, the fMRI experiment started.

The production lists were divided into 8 runs of 40 trials, each including 10 trials per condition, with as few verb repetitions as possible (per block 5–6 verbs were repeated once out of the 60 verbs presented (excluding fillers)). The comprehension lists were divided into 4 runs of 40 trials each. Production and comprehension runs alternated with two production runs always followed by a comprehension run. There were 12 acquisition runs in total. Each run lasted about 5 minutes. A fixation cross was presented for at least 800 ms before the picture screen was presented (Fig. 1). Participants had 5 seconds to produce the answer. This was followed by a blank screen for 200 ms. We jittered the onset of trials by 0–7500 ms (mean 1500 ms), by varying the length of presentation of the fixation cross. The order of conditions and length of jitter was based on design optimization for contrast detection, made with optseq2 (Dale 1999).

### fMRI Acquisition

MR data were acquired in a 3 T MAGNETOM PrismaFit MR scanner (Siemens AG, Healthcare Sector, Erlangen, Germany) using a 32-channel head coil. The MRI protocol included a T1-weighted MRI scan for anatomical reference and several fMRI scans. The T1-weighted scan was acquired in the sagittal orientation using a 3D MPRAGE sequence with the following parameters: repetition time (TR)/inversion time (TI) 2300/1100 ms, echo time (TE) 3 ms, 8° flip angle, field of view (FOV) 256 mm × 216 mm × 176 mm and a 1 mm isotropic resolution. Parallel imaging (iPAT = 2) was used to accelerate the acquisition resulting in an acquisition time of 5 min and 21 sec. Whole-brain functional images were acquired using a multi-band (accelerator factor of 3) multi-echo T2^*^-weighted sequence with the following parameters: TR 1500 ms, TEs 13.4/34.8/56.2, flip angle 75°, FOV 84 mm x 84 mm x 64 mm, voxel size 2.5 mm isotropic. Fieldmap images were also acquired to correct for distortions. We acquired 12 fMRI runs per participant.

### Data Analysis

#### Behavioural Analysis

Speech output in the production fMRI runs was analyzed for accuracy and response times. A Dutch native speaker rated the speech for accuracy. Speech was considered correct when the correct actors and determiners were used, the verb was inflected in the correct way, and the correct sentence structure was used. Self-corrections and word repetitions during hesitations were considered as errors. Speech onset and offset times were coded using Praat, after scanner noise removal. We analyzed onset and durations with linear mixed-effects models (Pinheiro and Bates 2000; Bates et al. 2015) and accuracy data using mixed-effects logit models (Jaeger 2008) with the *lme4* package (version 1.1–21, R version 3.6.2). We used the maximal effect structure that allowed for convergence (Barr et al. 2013). For accuracy, the model contained the factor Condition (C1, C2, C4) and by-participant and by-item (specifically, verbs) random intercepts. For onset and duration analysis, the model contained the factor Condition and by-participant random slopes for Condition and by-item random intercepts, with log-transformed onset and duration times.

#### fMRI Preprocessing

Preprocessing was performed using fMRIPprep 1.2.6–1 (Esteban et al. 2018; Esteban et al. 2018). The T1-weighted (T1w) image was corrected for intensity non-uniformity and skull-stripped. Brain surfaces were reconstructed using recon-all (FreeSurfer 6.0.1, Dale, et al. 1999). Spatial normalization to the ICBM 152 Nonlinear Asymmetrical template version 2009c (Fonov et al. 2009) was performed through nonlinear registration using brain-extracted versions of both T1w volume and template. Brain tissue segmentation of cerebrospinal fluid, white-matter and gray-matter was performed on the brain-extracted T1w using fast (FSL 5.0.9, Zhang et al. 2001).

For each of the BOLD runs per subject, the following preprocessing was performed. First, a reference volume and its skull-stripped version were generated using a custom methodology of fMRIPrep. A deformation field to correct for susceptibility distortions was estimated based on a field map that was co-registered to the BOLD reference, using a custom workflow of fMRIPrep. Based on the estimated susceptibility distortion, an unwarped BOLD reference was calculated for a more accurate co-registration with the anatomical reference. The BOLD reference was then co-registered to the T1w reference using bbregister (FreeSurfer). Co-registration was configured with nine degrees of freedom to account for distortions remaining in the BOLD reference. Head-motion parameters with respect to the BOLD reference (transformation matrices, and six corresponding rotation and translation parameters) were estimated before any spatiotemporal filtering using mcflirt (FSL 5.0.9, Jenkinson, et al. 2002). BOLD runs were slice-time corrected and resampled onto their original, native space by applying a single, composite transform to correct for head-motion and susceptibility distortions. Multi-echo combination was performed by estimating a T2^*^ map from the preprocessed BOLD by fitting to a monoexponential signal decay model with log-linear regression. For each voxel, the maximal number of echoes with reliable signal in that voxel were used to fit the model. The calculated T2^*^ map was then used to optimally combine preprocessed BOLD across echoes following the method described in (Posse et al. 1999). Estimation of motion artifacts using independent component analysis (ICA-AROMA, Pruim et al. 2015) was performed on the preprocessed BOLD on MNI space time-series after removal of non-steady state volumes and spatial smoothing with an isotropic, Gaussian kernel of 6 mm FWHM (full-width half-maximum). The AROMA noise-regressors were later used as confound regressors. The BOLD time-series were resampled to MNI152NLin2009cAsym standard space. Confounding time-series were calculated based on the preprocessed BOLD for framewise displacement (FD) and DVARS (following the definitions by Power et al. 2014). We excluded subjects that had FD values above 2.5 (these were also the subjects that showed highest mean FD and the largest number of volumes with FD values above 1). Additionally, a set of physiological regressors were extracted to allow for anatomical component-based noise correction (aCompCor, Behzadi, et al. 2007).

#### Motion-Related Correction

To prevent excessive motion artifacts due to speaking out loud, participants’ heads were secured in a pillow and a tape was attached across their foreheads to provide them with feedback in case of movement, which was shown to reduce motion (Krause et al. 2019). In addition, subjects with FD values above the voxel size were excluded. ICA-AROMA was used to estimate components related to motion that were later added as nuisance regressors together with motion parameters, FD, DVARS and aCompCor in the first-level design matrix.

### fMRI Analysis

#### Whole-Brain Analysis

We used the non-denoised preprocessed BOLD images in MNI152NLin2009cAsym standard space for first-level single-subject analysis. We applied spatial smoothing with an isotropic Gaussian kernel of 4 mm FWHM in SPM12 in Matlab2019a. For the production runs, we computed a general linear model (GLM) in SPM12 with the following condition regressors: correct trials for each of the four conditions, all incorrect trials, temporal derivative, and parametric modulations of speech onset times. For the comprehension runs, the GLM was identical except for the absence of an incorrect trial regressor and parametric modulations. The onset of each trial was set as the picture onset time, and trial duration was set as time until speech offset, hence accounting for differences in duration between individual stimuli and conditions. In addition, we added confound regressors that were computed in fMRIPrep. We included regressors for DVARS, Framewise Displacement, 6 aCompCor parameters and 6 motion parameters. Finally, we added the AROMA noise components computed in fMRIPrep as additional nuisance regressors, to perform non-aggressive denoising. Contrast images for the main effect of constituent size (with weights [−4 –1 5] based on constituent size of C1, C2 and C4, respectively), main effect of modality (production vs. comprehension) and interaction between constituent size and modality were computed for each participant. For the main effect of constituent size we selected a numerical linear contrast based on Pallier et al. (2011) that reflects activation with a linear increase according to the number of words integrated in a constituent: C1 = one content word per constituent, C2 = 2 content words, C4 = 4 content words. This led to a contrast with weights [−4 –1 5] after mean-centering. By contrasting the three conditions together, the results were less sensitive to other types of differences between individual conditions (e.g., the contrast C4 vs. C2 might be sensitive to verb argument structure differences). The contrast images were tested with a one-sample T-test at the group level following Henson (2015). We thresholded brain responses at the voxel-level at *p* = 0.001 *uncorrected*, and then used *p* = 0.05 Family-Wise Error corrected as the cluster threshold. We also ran a conjunction analysis to specifically look at the overlap between production and comprehension in the response to constituent size. To run the conjunction analysis, we created contrast images for the constituent size effect separately in production and comprehension, and then we entered them into a one-way ANOVA in SPM, with each as a separate cell. By defining separate contrasts for each, we could then run the conjunction analysis for the group-level contrast image of constituent structure in production and comprehension.

#### ROI Analysis

We took functional regions of interest (ROIs) based on the keyword “syntactic” in Neurosynth (https://www.neurosynth.org/, accessed on 08/01/2020, Yarkoni et al. 2011). This allowed us to select voxels that are reported to be active in multiple studies related to a key search word, here “syntactic”. We downloaded the active voxels with a z-score threshold of 9. This revealed two clusters, one in left IFG and one in left anterior and posterior middle temporal lobe. We extracted mean beta values per participant in each of these ROIs per condition (C1, C2, C4, in production and comprehension) relative to baseline using MarsBar (Brett et al. 2002) in SPM12. We then compared the beta weights in a mixed-effects model in R (version 3.6) using *lme4* (Bates et al. 2015), with constituent size (C1, C2, C4), modality (Production vs. Comprehension) and ROI (LIFG vs. LMTG) as factors. Deviation coding was used for factors modality and ROI, while a linear contrast with weights [−4 –1 5], as in the whole-brain analysis, was used for constituent size. We added by-participant random slopes for the interaction of ROI and modality and for the main effect of constituent size. We computed the contribution of factors using Type-III Wald tests in *car* (version 3.0–7, Fox et al. 2020) and pairwise comparisons for significant effects with the package *emmeans* (version 1.4.6, Lenth et al. 2020).

#### Exploratory Analysis: BOLD Peak Latency

As an additional exploratory analysis, we extracted BOLD time courses to determine whether the time to peak was influenced by region, modality and constituent size. To capture a delay in peak times, we used a finite impulse response (FIR) basis set as implemented in Marsbar in SPM12. This allowed us to get estimates of BOLD activity at each TR in the two ROIs for each participant. We then extracted BOLD peak times as the timepoint with highest amplitude between 1.5 and 9 s post stimulus onset for each participant. We ran a linear mixed-effect model with constituent size, modality and ROI as fixed effects, and by-participant random slopes for ROI. We used a linear contrast with weights [−4 –1 5] for constituent size, and deviation coding for modality and ROI. We computed the contribution of factors using *car* (version 3.0–7, Fox et al. 2020) and pairwise comparisons for significant effects with the package *emmeans* (version 1.4.6, Lenth et al. 2020).

## Results

### Behavioral Results

**Figure 2:**
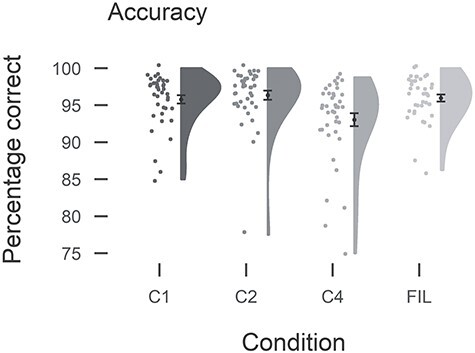
Individual and mean accuracy per condition. Black dots indicate mean with standard error of the mean. Gray dots represent individual participants’ mean.

**Figure 3:**
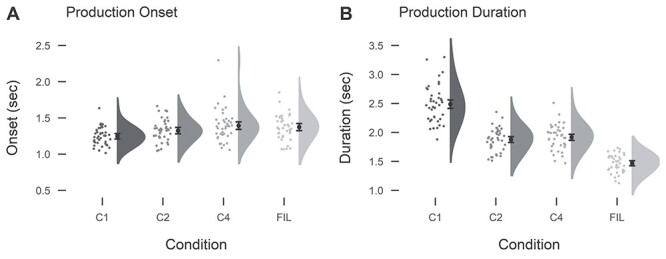
Onset (A) and duration (B) times per condition (of correct trials only). Black dots indicate mean with standard error of the mean. Gray dots represent individual participants’ mean.

Accuracy was generally high across participants and conditions (mean percentage correct: C1: 95.4, C2: 96.2, C4: 92.9, Fillers: 95.9; Fig. 2). There were slightly more errors in the C4 condition than in the C1 (β = 0.55, SE = 0.12, Z = 4.7, *p* < 0.001) and in the C2 conditions (β = 0.71, SE = 0.14, Z = 5.2, *p* < 0.001). Types of errors included using the wrong determiner (in Dutch, *het* is used with *meisje*-girl, and *de* with boy, man and woman; across all sentences for all participants, n = 117), the wrong actor (n = 170), a wrong verb or the correct verb in the wrong inflection/pronunciation (n = 105), the wrong condition (n = 119), not finishing within 5 seconds (n = 77), or other types of errors (n = 62). Unsurprisingly, onset times varied between conditions due to the characteristics of the conditions (mean onset times (in seconds): C1: 1.25, C2: 1.33, C4: 1.39, Fillers: 1.38, Fig. 3A). In particular, C1 elicited shorter reaction times than C2 (β = 0.06, SE = 0.01, *t* = 5.7, *p* < 0.001) and C4 (β = 0.11, SE = 0.01, *t* = 8.2, *p* < 0.001), as only the first verb had to be planned to initiate speech output. The other conditions, instead, required sentence planning, including subject (determiner and noun) as well as verb planning. The C4 condition elicited longer onset times than C2, too (β = 0.05, SE = 0.01, *t* = 5.1, *p* < 0.001). Similarly, duration times varied by condition (mean durations (in seconds): C1: 2.46, C2: 1.86, C4: 1.90, Fillers: 1.46; Fig. 3B). C1 production was characterized by the separate production of each lexical item, introducing pauses between words, and was thus characterized by the longest durations (vs. C2: β = 0.28, SE = 0.02, *t* = 16.6, *p* < 0.001; vs. C4: β = 0.27, SE = 0.02, *t* = 15.4, *p* < 0.001), while C2 and C4 did not differ in duration.

### Whole-Brain Analysis

**Figure 4:**
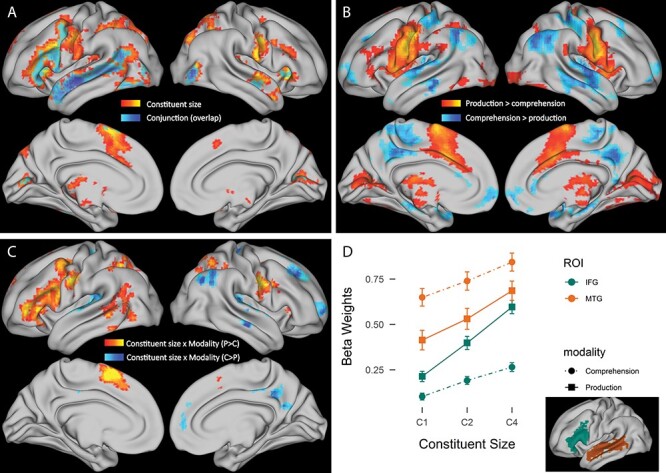
*Whole-brain and ROI results.*  **A**: orange: main effect of constituent size with a linear contrast for the three constituent sizes. Blue: conjunction analysis of production and comprehension constituent size effects representing areas active in both production and comprehension following the conjunction of null hypotheses (Friston et al. 2005). The blue area is superimposed on the corresponding cluster found as main effect of constituent size. **B**: whole-brain results for the main effect of modality. Orange: areas more active in production than comprehension. Blue: areas more active in comprehension than production. **C**: whole-brain results for the interaction between constituent size and modality. Orange: areas with larger response to constituent size in production than comprehension. Blue: areas with larger response to constituent size in comprehension than production. **D**: mean beta weights extracted from the predefined ROIs (depicted in figure), error bars represent standard error of the mean.

We focused on the main effects of constituent size in production and comprehension, and on the interaction between modalities (production vs. comprehension) and constituent size. For the main effect of constituent size, a large bilateral network centered around areas of the language network and the corresponding right hemisphere areas, with cerebellar and occipital activity, was found (Fig. 4A; Supplementary Table 1). We found a large left lateralized cluster including peaks in the left IFG, STG, MTG, temporal pole, precentral gyrus, postcentral gyrus, fusiform gyrus and superior parietal lobule. Similar right lateralized activity was found in a cluster in the temporal pole and IFG (*pars orbitalis*), a cluster in postcentral and precentral gyrus and a cluster in superior and middle temporal gyri, a cluster in superior parietal lobule and a cluster in the more posterior parts of the IFG (*pars triangularis* and *pars opercularis*). Additionally, we found clusters in the left and right supplementary motor area, in the left thalamus, left putamen, and right cerebellum.

To evaluate to what extent the activated network was overlapping between comprehension and production, we performed a conjunction analysis of the separate constituent size contrast for production and comprehension. This analysis revealed that in part the constituent size effect was reliably active in both modalities, with clusters in anterior and posterior MTG, LIFG, left precentral gyrus, left fusiform gyrus and right cerebellum (see Fig. 4A, Supplementary Table 1).

We also looked at the main effect of modality to understand if any areas were overall more active in production or comprehension (see Fig. 4B, Supplementary Table 2). We found bilateral frontal areas and parietal areas, as well as subcortical and cerebellar regions, to have larger activity in production than comprehension, partly reflecting articulatory requirements in production. Bilateral superior temporal areas were more engaged in comprehension, which was likely due to auditory processing. Bilateral angular gyrus, precuneus and superior frontal regions were also more engaged in comprehension.

A few areas responded differently to constituent size in production and comprehension (Fig. 4C, Supplementary Table 3). Areas that were more active with larger constituents in production were mainly left lateralized and included the LIFG (*pars triangularis*, *pars orbitalis* and *pars opercularis*), middle frontal gyrus, precentral gyrus, supplementary motor area, inferior and superior parietal lobule, supramarginal gyrus, angular gyrus, and posterior sections of the LMTG. Regions in the right hemisphere included precentral gyrus, postcentral gyrus, superior parietal lobule, supplementary motor area and cerebellum. A complementary network was more active in the comprehension of larger constituents, with peaks in bilateral Heschl’s gyrus, STG and temporal pole, and right hemisphere areas, including angular gyrus, precuneus, frontal pole and superior and middle frontal gyri.

### ROI Analysis: LIFG and LMTG

We extracted beta weights for the average activity in regions previously associated with syntactic effects to inspect patterns of activation for each condition in production and comprehension (Fig. 4D). We ran a linear mixed-effects model on the beta estimates that we extracted per condition per region. We found a main effect of constituent size (β = 0.027, SE = 0.001, *t* = 20.03, χ^2^ = 401.04, *p* < 0.0001), indicating that beta weights increased with larger constituent sizes. Pairwise comparisons indicated that beta weights for C4 were significantly larger than C2 and C1 in all modalities and ROIs (estimates > 0.10, *ts* > 8.2, *ps* < 0.0001). We also found a main effect of ROI (β = 0.18, SE = 0.02, *t* = 8.62, χ^2^ = 74.4, *p* < 0.0001), with larger beta estimates in LMTG than LIFG (estimate = 0.35, SE = 0.04, *t* = 8.52, *p* < 0.0001). The effect of ROI interacted with modality (β = 0.11, SE = 0.009, *t* = 11.45, χ^2^ = 131.03, *p* < 0.0001), since there was a larger difference in activity between ROIs in comprehension than in production (MTG—IFG, Production: estimate = 0.14, SE = 0.04, *t* = 2.97, *p* < 0.025; Comprehension: estimate = 0.56, SE = 0.04, *t* = 13.16, *p* < 0.0001). Importantly, there was a three-way interaction between constituent size, modality and ROI (β = 0.004, SE = 0.0009, *t* = 4.24, χ^2^ = 18.0, *p* < 0.0001). Inspection of the slopes for constituent size in each modality and ROI indicated that production elicited the steepest slope in the response to constituent size in the IFG: there was a larger slope difference between modalities in the IFG (Production—Comprehension: estimate = 0.023, SE = 0.003, *t* = 9.34, *p* < 0.0001) than in the MTG (Production—Comprehension: estimate = 0.009, SE = 0.003, *t* = 3.37, *p* = 0.005), and there was a slope difference between ROIs in production (IFG—MTG: estimate = 0.012, SE = 0.003, *t* = 4.5, *p* = 0.0001), but not in comprehension (MTG—IFG: estimate = 0.003, SE = 0.003, *t* = 1.47, *p* = 0.46). These results, therefore, show that: (i) larger constituent structures elicit higher activity in both regions and modalities, (ii) there is a stronger effect of constituent size in production than in comprehension, especially in LIFG, (iii) there is a higher response in LMTG than LIFG overall, and (iv) production elicits stronger activity than comprehension in the LIFG, while the opposite is the case in LMTG: more activity for comprehension than production.

### Exploratory Analysis: BOLD Peak Latency

**Figure 5:**
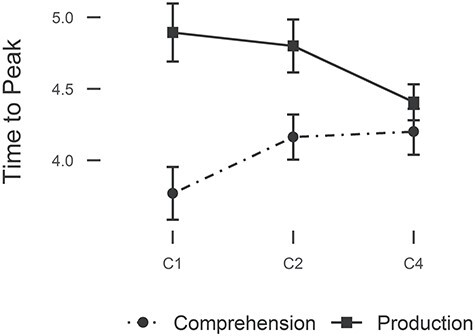
BOLD peak times averaged across participants and ROIs for each constituent size in production and comprehension. Error bars represent standard error of the mean.

We extracted BOLD times-to-peak for each condition to understand whether the regional and modality-specific effects highlighted by the ROI analysis were also characterized by BOLD time course differences. Pallier et al. (2011) had found that larger constituent sizes were associated with later peak times in the superior temporal sulcus and IFG, in line with the idea that activation is stronger towards the end of a constituent. A model with ROI, modality and constituent size as predictors for time-to-peak showed a main effect of modality (β = 0.33, SE = 0.06, *t* = 5.39, χ^2^ = 29.01, *p* < 0.0001), with comprehension peaking earlier than production (estimate = 0.66, SE = 0.12, *t* = 5.34, *p* < 0.0001). In addition, we found an interaction between modality and constituent size (β = 0.049, SE = 0.016, *t* = 3.002, χ^2^ = 9.01, *p* = 0.0027)^3^. Inspection of the slopes in the response to constituent size showed that comprehension elicited a positive slope, with larger constituent structures peaking later, while production elicited a negative slope, with larger constituent structures peaking earlier (Comprehension—Production: estimate = 0.098, SE = 0.033, *t* = 2.98, *p* = 0.0031) (Fig. 5). Therefore, the constituent size effect on peak latency that was found before (Pallier et al. 2011) seems to be dependent on modality, since in production an opposite pattern was found relative to comprehension.

## Discussion

We examined neural responses to the production and comprehension of utterances with increasing constituent size to clarify the neural correlates of sentence production and comprehension. We found that larger constituent sizes engaged areas traditionally part of the language network. These included inferior frontal regions, temporal and inferior parietal regions, mainly in the left hemisphere. Through a conjunction analysis, we confirmed that the LIFG and LMTG responded to constituent size in both comprehension and production. Increased syntactic complexity resulted in stronger activation in these areas. Moreover, we found a modality-specific dissociation, with production recruiting the LIFG more strongly than comprehension, and comprehension recruiting the LMTG more strongly than production. At the same time, the network was found to be differentially responsive to constituent size across modalities. While comprehension elicited similar responses to constituent size in LIFG and LMTG, in production the LIFG was more sensitive to constituent size than the LMTG. Finally, constituent size had opposite effects on BOLD peak latencies in comprehension and production: increasing constituent size elicited later peaks in comprehension but earlier peaks in production.

By demonstrating that the response to constituent size is largely shared between comprehension and production, we extend Pallier et al.’s constituent size effect (2011) to sentence production. Our results are in line with evidence associating sentence-level processes with left inferior frontal and temporal activation (Bornkessel-Schlesewsky et al. 2009; Shetreet et al. 2009; Segaert et al. 2012; Hagoort and Indefrey 2014; Blank et al. 2016; Henderson et al. 2016; Walenski et al. 2019; Indefrey 2018). Pallier et al. (2011) found that the LIFG and the posterior superior temporal sulcus were responsive to constituent size also with jabberwocky stimuli, while the ATL and the temporo-parietal junction only responded to stimuli with real words. All of these areas were responsive to constituent size also in the present study. Whether the IFG and the posterior temporal sulcus are sensitive to constituent size also with jabberwocky stimuli in production will have to be determined in future studies specifically designed to address the distinction between syntactic and semantic compositional processes. Finally, the activation delay for larger constituent structures was replicated here, but critically only in comprehension.

Our results, therefore, implicating both LIFG and LpMTG, as well as other areas of the language network, suggest that the inconsistent evidence for sentence production was due to low power in the single studies and in the meta-analyses (Walenski et al. 2019; Indefrey 2018). With 40 participants and a large number of trials per condition, we had enough power to detect effects in areas previously linked with sentence processing in comprehension. It is unlikely that the effects we found are reducible to the type of paradigm used to elicit sentence production, since other studies using picture descriptions or sentence reorganization paradigms also found activations in LIFG and/or LpMTG, but, critically, in an inconsistent way (e.g., pictures descriptions, Indefrey et al. 2001; Grande et al. 2012; Menenti et al. 2012; Segaert et al. 2012; sentence generation from words, Haller et al. 2005; Golestani et al. 2006; Collina et al. 2014). Moreover, although our paradigm was partly artificial in eliciting sentence production, it allowed us to cleanly manipulate constituent structure, ensuring consistent behavioral responses across participants. Previous studies used similar types of constrained elicitation paradigms or more constraining ones when more control over the production was required (cf. Matchin and Hickok 2016; Takashima et al. 2020; Matchin and Wood 2020).

Crucially, the conjunction analysis showed that production and comprehension engage largely overlapping areas in constituent structure building. An extensive network is engaged in sentence production that does not diverge from the one observed for comprehension in previous studies. The activation pattern, including left anterior and posterior MTG and LIFG is similar to the syntactic adaptation effects found across modalities in fMRI studies with repetition suppression (Menenti et al. 2011; Segaert et al. 2012). Our results do not provide information on whether verb-specific processing is also shared between production and comprehension, since the linear-contrast analysis avoided sensitivity to verb argument structure differences between sentences. Thus, these results confirm shared resources in sentence-level processes across modalities and provide no support for spatial segregation as a basis for distinct processes or representations. These findings, therefore, reconcile the previous inconsistent findings between sentence production and comprehension networks, as shown by meta-analyses (Walenski et al. 2019; Indefrey 2018), with the adaptation effects across modalities (Menenti et al. 2011; Segaert et al. 2012). Common neural resources provide a neural basis for views of shared linguistic representations and processes, such as retrieval and unification, between production and comprehension (Kempen 2000; Kempen et al. 2012; Dell and Chang 2014; Momma and Phillips 2018).

While the networks overlapped, there were differences in the degree to which each modality recruited core areas. In particular, we found that comprehension engaged the LMTG more than production, and production engaged the LIFG more than comprehension. This finding was consistent with the modality differences in the whole-brain results. Larger activity in the LMTG in comprehension was also found by Humphreys and Gennari (2014), and is likely due to the fact that the auditory input is processed in superior temporal areas with activity spreading in the temporal lobe, whereby the LpMTG might be involved in retrieval and integration of lexical, syntactic and semantic information, given its extensive connectivity patterns (Baggio and Hagoort 2011; Turken and Dronkers 2011; Binder 2017). The clusters showing more activity in production included not only the LIFG, but also more dorsal areas, extending to the precentral gyrus and the supplementary motor area. Together with the cerebellar activation, these latter areas are involved in articulation and motor planning (Price 2012; Basilakos et al. 2018).

The greater involvement of inferior frontal regions in production than comprehension is likely attributable to stronger sentence planning requirements, also reflected in the stronger effect of constituent size in the LIFG and to a smaller extent in the LpMTG in production than comprehension. In production, the syntactic structure of sentences needs to be fully and correctly computed in order to produce a well-formed utterance (Indefrey 2018; Garrett 1982; Garrett 1980). In comprehension, instead, inferring sentence meaning can often be done by retrieving word meanings and world knowledge, bypassing the need for a full syntactic analysis of the input (cf. good-enough processing, Ferreira et al. 2002). For instance, it has been shown that passive or object-relative sentences are sometimes interpreted in line with world knowledge but not necessarily in agreement with the syntactic structure (Ferreira 2003; Ferreira and Lowder 2016). Therefore, reduced sensitivity to constituent structure in comprehension may signal reduced syntactic processing in reaching the conceptual interpretation for these sentences. This fundamental difference between production and comprehension on the importance of “getting it right” may also explain the larger engagement of the default mode network in comprehension (in particular, right angular gyrus, right precuneus, right superior frontal gyrus and right frontal pole). We speculate that production disengaged the default mode network more than comprehension in responding to constituent size, due to the stronger requirements for accurate sentence planning (Raichle et al. 2001; Raichle and Snyder 2007).

The interaction effects between constituent size and modality cannot be reduced to task differences between modalities and in particular to the absence of an explicit task in comprehension. On the one hand, the constituent size effect in comprehension and the finding of larger comprehension activity in the LMTG confirm that participants processed the input even in the absence of a task (see Fig. 4D). On the other hand, the task requirements in production were very similar across levels of constituent size: what varied was the linguistic complexity of the output. Differences between modalities may instead show task effects, including cognitive control differences. However, as mentioned above, production is inherently a “task” as opposed to comprehension being more passive also in naturalistic situations. Task effects thus need not reflect spurious task differences due to the current design, but could be related to inherent differences in cognitive control between production and comprehension. Studies of spontaneous production may be able to address to what extent cognitive control is needed during naturalistic production as opposed to comprehension.

An additional dissociation in the response pattern for production and comprehension was found in the BOLD time courses. Production and comprehension elicited opposite profiles of response latencies in relation with constituent size. Larger structures were characterized by later peaks in comprehension, confirming previous evidence suggesting that larger structures take longer to be computed (Pallier et al. 2011). In contrast, larger structures elicited earlier peaks than smaller structures in production. This was likely due to planning differences between conditions. Reaction time analyses showed that onset times increased with constituent size, with C2 taking longer than C1, and C4 taking longer than C2. Since high-level processing can be initiated for the whole clause before speaking (Smith and Wheeldon 1999), it is likely that more extensive planning at the message or structural level took place in early stages for the more complex structures, inducing early peaks in BOLD activity. In contrast, in the conditions with smaller constituent size the structures to be computed were smaller and planning may have been in a word-by-word fashion interleaved with articulation, hence inducing sustained activity with later peaks. Since this was an exploratory analysis for which the stimuli and the design were not optimized *a priori*, future studies will need to clarify whether BOLD peak latencies in production are indeed influenced by planning scope and if the inverse relationship between onset times and production peak latencies holds with different stimuli and paradigms.

Overall, the current results are striking in showing how production and comprehension share resources but modulate them differently. Spatially, frontal and temporal regions are engaged in both modalities, but to different extents. Temporally, constituent size affects BOLD peak latencies in both modalities but in opposite directions. Rather than providing support for a distinction of core processes and representations between modalities (Meyer et al. 2016), this unbalanced sharing of resources reveals a “computational asymmetry” (Matchin and Hickok 2020) or “directional” differences (Pickering and Garrod 2013; Gambi and Pickering 2017). In production, linguistic processes map from higher to lower linguistic levels, i.e., meaning to phonology, and in comprehension from lower to higher linguistic levels, i.e., phonology to meaning (Pickering and Garrod 2013). This directional difference implies that the inputs and outputs of each modality are opposite in production and comprehension, which results in differences in recruitment patterns within the shared language network (Momma and Phillips 2018; Indefrey 2018), reflected not only in different regional levels of activity, but also in timing patterns.

In conclusion, the current results extend the constituent structure effect found in comprehension (Pallier et al. 2011) to production, and robustly show the involvement of both LpMTG and LIFG in constituent structure building in production, helping to clarify the inconsistencies in the previous studies on the neurobiology of language production. Additionally, the results confirm that the neural resources for sentence production and comprehension are largely overlapping, supporting accounts of shared representations between modalities. Finally, our results highlight modality-specific differences in regional and time course patterns that underline inevitable differences in the requirements of speaking and listening.

## Supplementary Material

SupplementaryMaterials_bhab287

## Data Availability

The data will be made available on the Donders Repository at https://data.donders.ru.nl/.

## Footnotes

1 The one provided is a literal translation of the Dutch sentence. A more natural translation would be “the boy is sleeping, the girl is talking”, since the present tense in Dutch is also used for continuous events.”

2 The example sentence provided “the boy hears that the girl claps” would also work with a bare infinitival complement construction (“the boy hears the girl clap”, “de jongen hoort het meisje klappen”), but many other verbs used in condition C4 do not support this construction (e.g., “klagen”, to complain, “dromen”, to dream). The word “dat” was thus required for all sentences in condition C4.

3 Since peak time extraction provided values in 1.5 sec resolution, we also ran a model with time (as well as constituent size, modality and ROI) as a predictor for percent signal change to ensure that extracted peak times were consistent with the time courses. The model returned a significant interaction between modality and time, and a three-way interaction between modality, constituent size and time, confirming the results of the peak time analysis.
