# Supplementary material for: Commonalities and Asymmetries in the Neurobiological Infrastructure for Language Production and Comprehension
Source: Cereb Cortex. 2021 Sep 7;32(7):1405–18. doi: 10.1093/cercor/bhab287 (PMC8971077; doi:10.1093/cercor/bhab287)
Supplement: SupplementaryMaterials_bhab287 [file supplementarymaterials_bhab287.doc]

**Supplementary Table 1**: fMRI whole-brain results for the main effect of constituent size and the conjunction analysis for production and comprehension constituent size effects.

| **Contrast** | **Cluster** | |  | **Peak Voxel (MNI Coordinates)** | | | | **Anatomical Location** |
| --- | --- | --- | --- | --- | --- | --- | --- | --- |
|  | ***p* (FWE-corrected)** | | **Size** | ***Z* score** | **x** | **y** | **z** |  |
| **Constituent size** | | 0 | 7656 | Inf | -48 | 18 | 24 | LIFG (*pars triangularis*) |
|  |  | |  | 7.66 | -58 | -2 | -13 | Superior Temporal Gyrus |
|  |  | |  | 7.42 | -28 | 28 | 0 | Left Anterior Insula |
|  |  | |  | 7.28 | -51 | 20 | -6 | LIFG (*pars orbitalis*) |
|  |  | |  | 7.28 | -51 | -14 | -8 | LMTG |
|  |  | |  | 6.79 | -51 | 16 | -23 | Temporal Pole |
|  |  | |  | 6.52 | -46 | 8 | 37 | Left Precentral Gyrus |
|  |  | |  | 6.46 | -46 | -40 | -20 | Left Inferior Temporal Gyrus |
|  |  | |  | 6.41 | -26 | -77 | 32 | Left Middle Occipital Gyrus |
|  |  | |  | 6.32 | -44 | -57 | -16 | Left Fusiform Gyrus |
|  |  | |  | 6.27 | -54 | -10 | 40 | Left Postcentral Gyrus |
|  |  | |  | 6.21 | -61 | -57 | 14 | LSTG |
|  |  | |  | 6.15 | -38 | -50 | 50 | Left Inferior Parietal Lobule |
|  |  | |  | 6.11 | -54 | 33 | 14 | LIFG (*pars triangularis*) |
|  |  | |  | 5.07 | -11 | -77 | 57 | Left Superior Parietal Lobule |
|  |  | |  | 4.79 | -28 | 0 | 67 | Left Middle Frontal Gyrus |
|  | 0 | | 1953 | Inf | 14 | -70 | -28 | Right Cerebellum |
|  |  | |  | 6.59 | -16 | -67 | -18 | Left Cerebellum |
|  | 0 | | 642 | 6.81 | -4 | 8 | 62 | Left Supplementary Motor Area |
|  | 0 | | 440 | 6.69 | 32 | 30 | 0 | RIFG (*pars orbitalis*) |
|  |  | |  | 5.52 | 49 | 18 | -13 | Right Temporal Pole |
|  | 0 | | 204 | 6.12 | 49 | -74 | 2 | Right MTG |
|  | 0 | | 194 | 5.72 | 64 | -4 | -3 | Right STG |
|  |  | |  | 4.89 | 49 | -14 | -13 | Right MTG |
|  | 0 | | 388 | 5.69 | 54 | -10 | 40 | Right Precentral Gyrus |
|  |  | |  | 5.39 | 66 | -7 | 14 | Right Postcentral Gyrus |
|  | 0 | | 422 | 5.64 | -8 | -82 | 10 | Left Calcarine Sulcus |
|  |  | |  | 5.31 | 9 | -72 | 12 | Right Calcarine Sulcus |
|  | 0 | | 61 | 5.55 | -34 | -27 | 10 | Left Heschl's Gyrus |
|  | 0 | | 455 | 5.3 | -11 | -30 | -13 | Brainstem |
|  |  | |  | 5.08 | -14 | -2 | 20 | Left Caudate |
|  |  | |  | 4.99 | -11 | -17 | 10 | Thalamus |
|  |  | |  | 4.94 | 6 | 6 | 0 | Right Caudate |
|  | 0 | | 707 | 5.22 | 29 | -80 | 40 | Right Superior Occipital Gyrus |
|  |  | |  | 5.07 | 34 | -57 | 47 | Right Angular Gyrus |
|  |  | |  | 5.02 | 19 | -60 | 60 | Right Superior Parietal Lobule |
|  | 0.032 | | 28 | 5.18 | -46 | -57 | -26 | Left Cerebellum |
|  | 0 | | 98 | 5.03 | 36 | -22 | 10 | Right Insula |
|  | 0.001 | | 52 | 4.69 | 32 | -10 | 67 | Right Superior Frontal Gyrus |
|  | 0 | | 76 | 4.68 | -31 | -12 | -6 | Left Putamen |
|  | 0 | | 264 | 4.53 | 56 | 18 | 32 | Right IFG (*pars opercularis*) |
|  |  | |  | 4.41 | 42 | 23 | 22 | Right IFG (*pars triangularis*) |
|  | 0 | | 67 | 4.53 | -8 | 58 | 37 | Left Superior Frontal Gyrus |
| **Conjunction** | 0 | | 2757 | 7.73 | -54 | -12 | -8 | LMTG |
|  |  | |  | 6.74 | -58 | 16 | 17 | LIFG (*pars opercularis*) |
|  |  | |  | 6.27 | -48 | 18 | -18 | Left Temporal Pole |
|  |  | |  | 6.07 | -46 | 28 | -6 | LIFG (*pars orbitalis*) |
|  |  | |  | 5.91 | -46 | -57 | 20 | Left Angular Gyrus |
|  |  | |  | 5.58 | -56 | 33 | 7 | LIFG (*pars triangularis*) |
|  |  | |  | 5.38 | -41 | -42 | -23 | Left Fusiform Gyrus |
|  |  | |  | 4.66 | -44 | 8 | 30 | Left Precentral Gyrus |
|  |  | |  | 4.54 | -46 | -84 | 4 | Left Middle Occipital Gyrus |
|  | 0 | | 344 | 7.01 | 19 | -80 | -40 | Right Cerebellum |
|  | 0 | | 117 | 5.47 | 49 | -74 | 0 | Right MTG |
|  | 0 | | 69 | 4.7 | -6 | -80 | 12 | Left Calcarine Sulcus |
|  | 0 | | 59 | 4.22 | -28 | -74 | 32 | Left Middle Occipital Gyrus |
|  |  | |  | 3.6 | -24 | -82 | 44 | Left Superior Parietal Lobule |
|  | 0.01 | | 37 | 4.11 | -4 | 8 | 70 | Left Supplementary Motor Area |
|  | 0.001 | | 58 | 4.11 | 12 | -74 | 10 | Right Calcarine Sulcus |
|  | 0.001 | | 57 | 4.07 | 36 | 23 | -3 | Right Insula |
|  | 0.001 | | 56 | 4.05 | 54 | -4 | -13 | Right STG |

**Supplementary Table 2**: fMRI whole-brain results for the main effect of modality.

| **Contrast** | **Cluster** |  | **Peak Voxel (MNI Coordinates)** | | | | **Anatomical Location** |
| --- | --- | --- | --- | --- | --- | --- | --- |
|  | ***p* (FWE-corrected)** | **Size** | ***Z* score** | **x** | **y** | **z** |  |
| **Production > Comprehension** | 0 | 14691 | Inf | -51 | -12 | 40 | Left Postcentral Gyrus |
|  |  |  | Inf | 49 | -7 | 30 | Right Postcentral Gyrus |
|  |  |  | Inf | 22 | -64 | -23 | Right Cerebellum |
|  |  |  | Inf | -14 | -17 | 4 | left Thalamus |
|  |  |  | Inf | -16 | -67 | -18 | Left Cerebellum |
|  |  |  | Inf | -31 | -17 | 0 | Left Putamen |
|  |  |  | Inf | 32 | -14 | -3 | Right Putamen |
|  |  |  | Inf | 14 | -17 | 7 | Right Thalamus |
|  |  |  | 7.57 | 39 | 6 | 4 | Right Insula |
|  |  |  | 7.41 | -34 | -12 | 20 | Left Insula |
|  |  |  | 6.54 | 22 | 0 | 17 | Right Caudate |
|  |  |  | 6.45 | -54 | 6 | 44 | Left Precentral Gyrus |
|  |  |  | 5.83 | 14 | -62 | 7 | Right Calcarine Sulcus |
|  |  |  | 5.83 | -11 | -70 | 7 | Left Calcarine Sulcus |
|  |  |  | 5.59 | -41 | 23 | 0 | LIFG (*pars triangularis*) |
|  |  |  | 4.87 | 32 | -92 | -10 | Right Inferior Occipital Gyrus |
|  |  |  | 4.79 | -54 | -54 | -16 | Left Inferior Temporal Gyrus |
|  |  |  | 4.73 | 46 | -10 | 57 | Right Precentral Gyrus |
|  |  |  | 4.7 | 34 | -57 | -16 | Right Fusiform Gyrus |
|  |  |  | 4.3 | 39 | 8 | 27 | Right IFG (*pars opercularis*) |
|  |  |  | 4.22 | -41 | -64 | -6 | Left Inferior Occipital Gyrus |
|  |  |  | 4.03 | -38 | 40 | 24 | Left Middle Frontal Gyrus |
|  |  |  | 3.91 | -14 | 18 | -13 | Left Medial Orbital Gyrus |
|  |  |  | 3.8 | -31 | 8 | 27 | LIFG (*pars opercularis*) |
|  |  |  | 3.78 | 19 | -44 | -6 | Right Parahippocampal gyrus |
|  |  |  | 3.78 | -28 | -72 | -10 | Left Fusiform Gyrus |
|  |  |  | 3.19 | -64 | -30 | 34 | Left Supramarginal Gyrus |
|  | 0 | 2087 | Inf | 4 | 16 | 37 | Right Middle Cingulate Cortex |
|  |  |  | Inf | 4 | 0 | 67 | Right Supplementary Motor Area |
|  |  |  | 7.06 | -4 | 10 | 57 | Left Supplementary Motor Area |
|  |  |  | 5.27 | -4 | -4 | 40 | Left Middle Cingulate Cortex |
|  | 0 | 128 | 7.39 | 19 | -30 | 64 | Right Precentral Gyrus |
|  | 0 | 128 | 6.95 | -21 | -32 | 62 | Left Postcentral Gyrus |
|  | 0 | 109 | 5.39 | -21 | -97 | -10 | Left Inferior Occipital Gyrus |
|  | 0 | 517 | 4.91 | -26 | -72 | 44 | Left Inferior Parietal Lobule |
|  | 0.002 | 53 | 4.76 | 62 | -34 | 22 | Right Supramarginal Gyrus |
|  | 0 | 63 | 4.02 | -58 | -44 | 27 | Left Supramarginal Gyrus |
|  | 0.01 | 39 | 3.94 | 22 | -67 | 50 | Right Superior Parietal Lobule |
|  | 0.029 | 32 | 3.87 | -41 | -87 | 2 | Left Middle Occipital Gyrus |
| **Comprehension > Production** | 0 | 1907 | Inf | 46 | -14 | 2 | Right Transverse Temporal Gyrus |
|  |  |  | 6.43 | 64 | -10 | -3 | Right STG |
|  |  |  | 6.09 | 49 | -10 | -16 | Right MTG |
|  |  |  | 5.87 | 39 | -22 | 14 | Right Heschl's Gyrus |
|  |  |  | 5.8 | 39 | 18 | -26 | Right Superior Temporal Pole |
|  |  |  | 5.38 | 26 | -20 | -16 | Right Hippocampus |
|  |  |  | 3.8 | 16 | -32 | -13 | Right Parahippocampal gyrus |
|  | 0 | 2110 | Inf | -46 | -14 | 2 | Left Transverse Temporal Gyrus |
|  |  |  | 6.83 | -58 | -27 | 12 | Left STG |
|  |  |  | 6.39 | -54 | -62 | 42 | Left Angular Gyrus |
|  |  |  | 6.25 | -61 | -34 | -10 | Left MTG |
|  |  |  | 5.07 | -41 | 16 | -26 | Left Superior Temporal Pole |
|  |  |  | 4.58 | -48 | -24 | -18 | Left Inferior Temporal Gyrus |
|  | 0 | 615 | 6.98 | -18 | -84 | -30 | Left Cerebellum |
|  | 0 | 198 | 6.83 | -18 | -17 | 30 | Left Postcentral Gyrus |
|  | 0 | 1849 | 6.68 | -1 | -44 | 40 | Left Precuneus |
|  |  |  | 5.87 | -4 | -24 | 50 | Left Paracentral Lobule |
|  |  |  | 5.76 | 14 | -52 | 37 | Right Precuneus |
|  | 0 | 525 | 6.31 | 42 | -32 | 60 | Right Postcentral Gyrus |
|  | 0 | 627 | 6.25 | -1 | 53 | -6 | Left Medial Frontal Cortex |
|  | 0 | 911 | 6.2 | 56 | -54 | 40 | Right Angular Gyrus |
|  | 0 | 119 | 6.18 | 9 | -47 | -46 | Right Cerebellum |
|  | 0 | 408 | 6.16 | 14 | -82 | -28 | Right Cerebellum |
|  | 0 | 351 | 6.02 | -36 | 63 | 2 | Left Lateral Orbital Cortex |
|  | 0 | 131 | 5.55 | -26 | 36 | 50 | Left Middle Frontal Gyrus |
|  | 0 | 264 | 5.44 | 29 | 66 | 10 | Right Superior Frontal Cortex |
|  | 0 | 250 | 5.36 | 42 | 23 | 50 | Right Middle Frontal Gyrus |
|  | 0 | 311 | 5.31 | -36 | -40 | 64 | Left Postcentral Gyrus |
|  | 0 | 76 | 5.3 | 2 | 38 | 2 | Right Anterior Cingulate Cortex |
|  |  |  | 4.87 | -1 | 28 | 14 | Left Anterior Cingulate Cortex |
|  | 0 | 86 | 4.98 | -21 | -27 | -18 | Left Parahippocampal Gyrus |
|  | 0.029 | 32 | 4.1 | 36 | 38 | -10 | Right Lateral Orbital Gyrus |

**Supplementary Table 3**: fMRI whole-brain results for the interaction between modality and constituent size, with clusters showing increased activity for larger constituents in production or comprehension.

| **Contrast** | **Cluster** |  | **Peak Voxel (MNI Coordinates)** | | | | **Anatomical Location** |
| --- | --- | --- | --- | --- | --- | --- | --- |
|  | ***p* (FWE-corrected)** | **Size** | ***Z* score** | **x** | **y** | **z** |  |
| **Interaction Production > Comprehension** | 0 | 2307 | 6.41 | -51 | 20 | -6 | LIFG (*pars orbitalis*) |
|  |  |  | 6.35 | -51 | 10 | 32 | Left Precentral Gyrus |
|  |  |  | 5.83 | -44 | 43 | 2 | LIFG (*pars triangularis*) |
|  |  |  | 5.67 | -28 | -7 | 50 | Left Middle Frontal Gyrus |
|  |  |  | 5.29 | -58 | 13 | 12 | LIFG (*pars opercularis*) |
|  | 0 | 1108 | 5.53 | -26 | -54 | 42 | Left Inferior Parietal Lobule |
|  |  |  | 5.51 | -34 | -64 | 57 | Left Superior Parietal Lobule |
|  |  |  | 5.49 | -26 | -74 | 37 | Left Angular Gyrus |
|  |  |  | 4.97 | -51 | -50 | 54 | Left Supramarginal Gyrus |
|  | 0 | 1032 | 7.74 | 12 | -82 | -40 | Right Cerebellum |
|  | 0 | 388 | 7.29 | -4 | 16 | 57 | Left Supplementary Motor area |
|  |  |  | 3.98 | 12 | 10 | 50 | Right Supplementary Motor Area |
|  | 0 | 271 | 5.52 | 52 | -4 | 32 | Right Postcentral Gyrus |
|  | 0 | 783 | 5.47 | -61 | -52 | 10 | LMTG |
|  |  |  | 5.37 | -56 | -67 | 20 | Left Angular Gyrus |
|  |  |  | 4.14 | -51 | -74 | -10 | Left Inferior Occipital Gyrus |
|  | 0 | 277 | 5.28 | 29 | -54 | 42 | Right Inferior Parietal Sulcus |
|  |  |  | 4.62 | 32 | -62 | 62 | Right Superior Parietal Lobule |
|  |  |  | 4.16 | 42 | -34 | 44 | Right Supramarginal Gyrus |
|  | 0 | 93 | 4.77 | 44 | 8 | 30 | Right Precentral Gyrus |
|  |  |  | 3.72 | 56 | 18 | 32 | Right IFG (*pars opercularis*) |
|  | 0.001 | 50 | 4.74 | 36 | 26 | 2 | Right Anterior Insula |
|  | 0.003 | 44 | 4.72 | 2 | -62 | -38 | Right Cerebellum |
|  | 0.002 | 46 | 4.5 | -54 | -50 | -16 | Left Inferior Temporal |
|  | 0 | 67 | 4.36 | -11 | 56 | 34 | Left Superior Frontal Gyrus |
|  |  |  | 3.62 | -8 | 46 | 47 | Left Superior Medial Frontal Gyrus |
|  | 0 | 94 | 4.33 | 34 | -70 | 34 | Right Middle Occipital Gyrus |
|  |  |  | 3.47 | 26 | -72 | 54 | Right Superior Parietal Lobule |
|  | 0.02 | 31 | 4.04 | -16 | -67 | -20 | Left Cerebellum |
| **Interaction Comprehension > Production** | 0 | 534 | 5.57 | -38 | -24 | 7 | Left Heschl's Gyrus |
|  |  |  | 4.42 | -36 | -32 | 22 | Left Posterior Insula |
|  |  |  | 4.35 | -58 | -17 | 7 | Left Superior Temporal Gyrus |
|  |  |  | 4.23 | -48 | 8 | -10 | Left Superior Temporal Pole |
|  | 0 | 350 | 5.57 | 44 | -14 | 0 | Right STG |
|  |  |  | 4.61 | 36 | -24 | 14 | Right Heschl's Gyrus |
|  | 0 | 374 | 5.51 | 24 | 36 | 42 | Right Superior Frontal Gyrus |
|  |  |  | 4.12 | 42 | 30 | 42 | Right Middle Frontal Gyrus |
|  | 0 | 371 | 5.27 | 59 | -54 | 40 | Right Inferior Parietal Lobule |
|  |  |  | 4.9 | 44 | -64 | 32 | Right Angular Gyrus |
|  | 0 | 168 | 5.14 | 66 | -17 | -6 | Right MTG |
|  | 0.039 | 27 | 4.6 | 14 | -27 | 64 | Right Precentral Gyrus |
|  | 0.001 | 53 | 4.59 | -31 | 38 | 40 | Left Middle Frontal Gyrus |
|  | 0 | 179 | 4.56 | 26 | 66 | 4 | Right Frontal Pole |
|  |  |  | 3.64 | 2 | 63 | 4 | Right Superior Medial Frontal Gyrus |
|  | 0 | 100 | 4.52 | 9 | -44 | 37 | Right Precuneus |
|  | 0.001 | 51 | 4.5 | 6 | -57 | 24 | Right Precuneus |
|  | 0 | 70 | 4.48 | 9 | -24 | 42 | Right Middle Cingulate Gyrus |
|  |  |  | 3.66 | 9 | -37 | 52 | Right Precuneus |
|  | 0 | 95 | 4.06 | 14 | 53 | 0 | Right Superior Medial Frontal Gyrus |
